# Alkene Cleavage Catalysed by Heme and Nonheme Enzymes: Reaction Mechanisms and Biocatalytic Applications

**DOI:** 10.1155/2012/626909

**Published:** 2012-07-03

**Authors:** Francesco G. Mutti

**Affiliations:** Department of Chemistry, Organic and Bioorganic Chemistry, University of Graz, Heinrichstrasse 28, 8010 Graz, Austria

## Abstract

The oxidative cleavage of alkenes is classically performed by chemical methods, although they display several drawbacks. Ozonolysis requires harsh conditions (−78°C, for a safe process) and reducing reagents in a molar amount, whereas the use of poisonous heavy metals such as Cr, Os, or Ru as catalysts is additionally plagued by low yield and selectivity. Conversely, heme and nonheme enzymes can catalyse the oxidative alkene cleavage at ambient temperature and atmospheric pressure in an aqueous buffer, showing excellent chemo- and regioselectivities in certain cases. This paper focuses on the alkene cleavage catalysed by iron cofactor-dependent enzymes encompassing the reaction mechanisms (in case where it is known) and the application of these enzymes in biocatalysis.

## 1. Introduction

The oxidative cleavage of alkenes is a widely employed method in synthetic chemistry, particularly to introduce oxygen functionalities into molecules, remove protecting groups, and degrade large molecules. Moreover, the synthesis of a large amount of bioactive compounds involves the alkene cleavage as a key step. Ozonolysis is the most employed chemical method for cleaving alkenes since it is considered the most efficient and cleanest. However, the ozonolysis requires harsh conditions such as low temperature (ca. −78°C), hence imposing the use of a special equipment (e.g., ozoniser) and reducing reagents in molar amounts during the workup [1]. Furthermore, safety hazards complicate this reaction on large scale, and serious accidents from explosion have been reported [2, 3]. Alternative protocols envisage the use of poisonous heavy metals such as Cr, Os, or Ru which are plagued by mediocre yields and selectivities [4–6]. Conversely, enzymes can activate the most innocuous oxidant, that is, molecular oxygen, and catalyse the alkene cleavage at ambient temperature and atmospheric pressure in aqueous buffer. Besides, in certain cases enzymes are capable to cleave olefinic functionalities in high chemo- and regioselective fashion allowing biocatalysis to compete with chemical methods [7–9]. 

Otherwise, the rising popularity of natural products during the last decade has triggered off remarkable research activities regarding the use of biocatalysis for the production of flavour compounds [10]. In fact, products derived from the bioprocess of natural substrates (i.e., using wild-type microorganisms or isolated enzymes thereof) are defined as natural. The tag natural was one of the main reasons for seeking biochemical routes to high-priced natural flavours such as vanillin, and nowadays biocatalysis constitutes a convenient alternative to synthesise them.

This paper focuses on the alkene cleavage catalysed by iron cofactor-dependent enzymes encompassing the reaction mechanisms (in case where it is known) and the application of these enzymes in biocatalysis. In the first part heme peroxidases are examined, for which the alkene cleavage is principally a promiscuous activity. In the second part heme and nonheme oxygenases are discussed, for which a more detailed survey concerning the reaction mechanisms is available in literature. 

## 2. Alkene Cleavage by Heme Peroxidases

**Scheme 1:**
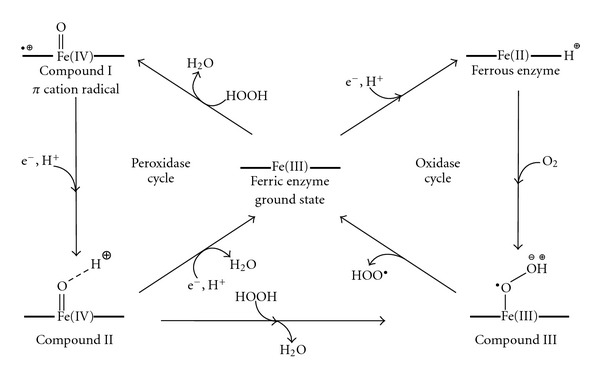
The five oxidation states of peroxidases.

Peroxidases are ubiquitously found in microorganisms, plants, and animals; these enzymes are named after their natural sources such as horseradish peroxidase, lactoperoxidase, myeloperoxidase or after their natural substrates such as cytochrome *c*, chloroperoxidase, and lignin peroxidase. The principally studied peroxidases are heme enzymes, hence possessing a ferric protoporphyrin IX (protoheme) as the prosthetic group [11]. Heme-containing enzymes participate in a strikingly diverse range of chemistry; yet all biological oxidation reactions catalysed by these enzymes involve very similar high oxidation state intermediates whose reactivity is modulated by the protein environment (Scheme 1) [12, 13].

Consequently, peroxidases are extremely promiscuous enzymes since they catalyse diverse chemical transformations such as peroxidase, peroxygenase, and oxidase reactions, acting on a vast array of substrates including phenols, aromatic and alkyl amines, NAD(P)H, H_2_O_2_, peracids, alkenes, thioethers, and aldehydes [11]. Particularly, it was shown that peroxidases catalyse the aerobic oxidation of aldehydes to yield carbonylic products via an enol tautomer, therefore postulating the formation of a dioxetane intermediate (*α*-oxidation) [14]. This mechanism somehow resembles the alkene cleaving activity, which has been observed as a side reaction in various peroxidases. Depending on the enzyme and the specific substrate involved, few speculative reaction mechanisms for the aerobic alkene cleavage of alkenes leading to aldehydes have been proposed, albeit a catalytic cycle has not been proved to date. A survey of alkene cleavage reactions catalysed by peroxidases is presented in the following sections.

### 2.1. Chloroperoxidase

**Scheme 2:**
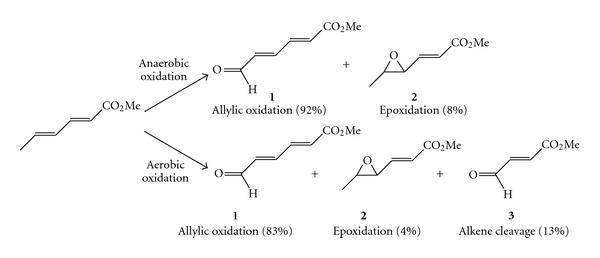
Aerobic and anaerobic CPO-catalysed oxidation of methyl-(2E,4E)-hexadienoate.

Chloroperoxidase (CPO) was isolated from *Caldariomyces fumago* [15, 16], and it is one of the most versatile and promising heme enzymes for synthetic applications [17–20]. For instance, various transformations typical for catalases and cytochrome P-450 monooxygenases are also catalysed by CPO [21]. Furthermore, the enzyme catalyses halide-dependent as well as halide-independent reactions acting on various substrates and using hydrogen peroxide or organic peroxides as oxygen source [11]. Other studies have shown that CPO catalyses the epoxidation of a number of functionalised or unfunctionalised olefins with high degree of enantio- and diastereoselectivity [22–24]. Epoxidation was often accompanied by the formation of aldehydes as well as by allylic hydroxylation [18]. Bougioukou and Smonou reported the alkene cleaving activity for the oxidation of conjugated dienoic esters employing CPO and *tert*-butyl-hydroperoxide (*t*BHP) as terminal oxidant [25, 26]. The reactions were carried out on alkenes with *cis-* and *trans-*configuration under anaerobic and aerobic conditions. In absence of molecular oxygen only two reactions occurred: the allylic oxidation and the epoxidation to give compounds **1** and **2**, respectively (Scheme 2). Both reactions proceeded with a high degree of regioselectivity, since the C=C bond proximal to the ester moiety was not converted. Surprisingly, the reaction performed under aerobic conditions furnished an additional aldehyde **3** as a minor product (13%) via the cleavage of the terminal C=C double bond. Also in this case, the alkene cleavage proceeded with perfect regioselectivity.

**Scheme 3:**
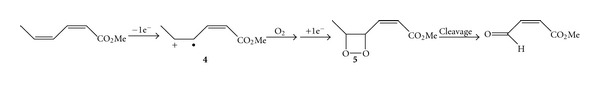
Proposed mechanism for the aerobic alkene cleavage catalysed by CPO.

Moreover, the relative amounts of the products depended on the stereochemistry of the double bond, since the allylic aldehyde was the preferred product starting from *trans-*dienes. Nevertheless, the same reaction performed on methyl-(2Z,4Z)-hexadecanoate furnished the cleaved alkene as the main product (38%), followed by the epoxide (35%) and the allylic aldehyde (27%). The proposed explanation for the formation of all products, included 3 under aerobic but not anaerobic conditions, involves the formation of an intermediate radical cation (**4**). This is probably generated by a direct electron transfer from the substrate to the formally oxoiron (V) centre (compound I, see Scheme 1) formed in the CPO catalytic cycle [12, 27]. Alternatively a similarly generated *t*-BuOO^•^ radical may act as a mediator, hence abstracting the electron from the *π* C=C bond of the substrate. Finally, the radical intermediate **4** can react with dioxygen, leading to the cleavage product via a dioxetane intermediate (**5**) (Scheme 3).

### 2.2. Horseradish Peroxidase

Horseradish peroxidase (HRP) is the most studied and well-characterised peroxidase, whose crystal structure [28] and catalytic pathway have been elucidated at high resolution [12]. HRP catalyses the oxidation of phenols, anilines, and a variety of other electron-rich compounds at the expense of H_2_O_2_ and alkyl hydroperoxides [11]. Ortiz De Montellano and Grab observed the oxidation of styrene to styrene oxide and benzaldehyde (molar ratio 4 : 1) upon incubating the substrates with HRP, H_2_O_2_, and 4-methylphenol as cooxidant [29]. By removing one of the compounds, styrene oxidation was not detected anymore. Besides, ^18^O_2_ and H_2_^18^O labelling studies have shown that the reaction mechanism for the formation of styrene oxide involves a radical intermediate which originates from the cooxidant. In the same work an explanation for the formation of benzaldehyde was not provided.

In another study, some HRP mutants showed an enhanced activity compared to the wild-type enzyme for the epoxidation of styrene as well as *cis-β*-methyl-styrene and *trans-β*-styrene in absence of any cooxidant [30]. Whilst a significant amount of benzaldehyde was produced, also in this case the catalytic cycle leading to this product was not elucidated. The increased activity stemmed probably from the improved accessibility of the substrate into the catalytic site, due to the replacement of the sterically hindering Phe-41 residue, located close to the heme centre, with a smaller amino acid such as leucine or threonine. 

**Scheme 4:**
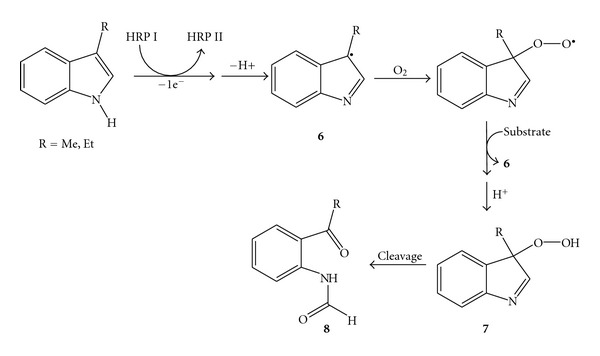
Proposed mechanism for the cleavage of indoles catalysed by HRP under aerobic conditions.

Oxidative cleavage of 3-methyl-indole and 3-ethyl-indole to the corresponding ring-opened *ortho*-acyl formanilides and oxindoles was carried out on a 50 mg scale using HRP under aerobic conditions [31]. Interestingly for a practical application, the molar ratio substrate HRP was 10000 : 1. The radical oxidation was initiated by the addition of a catalytic amount of H_2_O_2_, required to generate compounds I and II (Scheme 1) from the resting state of the HRP; however, H_2_O_2_ concentration was kept below 30 *μ*M to avoid enzyme deactivation. Under aerobic conditions the main product was the carbonylic compound from the alkene cleavage, whereas under anaerobic conditions the radical intermediate completely polimerised. A mechanism for the aerobic oxidative cleavage of indole was finally proposed, involving hydrogen abstraction by HRP compound I or II (**6**), interaction with dioxygen to lead the hydroperoxide (**7**), and final rearrangement to afford the carbonylic product (**8**) (Scheme 4).

**Scheme 5:**
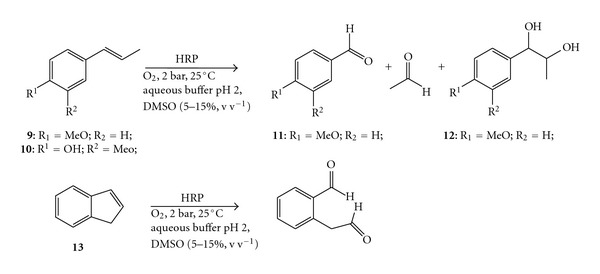
Alkene cleavage of *trans*-anethole (**9**), isoeugenol (**10**), and indene (**13**) catalysed by HRP at 2-bar dioxygen pressure and ambient temperature.

Mutti et al. have recently shown that some peroxidases (i.e., horseradish peroxidase, lignin peroxidase, and *Coprinus cinereus *peroxidase) catalyse the cleavage of a C=C double bond adjacent to an aromatic moiety for selected substrates at the expense of molecular oxygen and at an acidic pH (Scheme 5) [32]. Among the three active peroxidases, HRP turned out to be the most active when an equal concentration of enzyme was employed. A thorough study of the reaction showed that the highest activity was obtained at ambient temperature, at pH 2, and at 2 bars of pure dioxygen pressure. Addition of DMSO as cosolvent up to 15% v v^−1^ increased the conversion, probably due to the improved solubility of the substrates in the aqueous reaction medium, while a further addition of DMSO led to a progressive decline of the enzymatic activity. Using *trans*-anethole as substrate (**9**) (6 g L^−1^) and HRP at low catalyst loading (3 mg, equal to 0.2–0.3 mol%), quantitative conversion was achieved within 24 h. The main product was *para*-anisaldehyde (**11**) (i.e., 92% chemoselectivity), whereas the side product accounted completely for the vicinal diol (**12**). The substrate spectrum was quite narrow, since only other two substrates, that is, isoeugenol (**10**) and indene (**13**), could be cleaved by HRP with 12% and 72% conversion, respectively. 

### 2.3. Myeloperoxidase and *Coprinus cinereus*  Peroxidase

Myeloperoxidase (MPO) and *Coprinus cinereus* peroxidase (CiP) catalyse the enantioselective epoxidation of styrene and a number of substituted derivates in moderate yield [33]. Additionally, as a consequence of the C_*α*_=C_*β*_ double bond cleavage of variously substituted styrene precursors, both MPO and CiP form significant amounts of substituted benzaldehydes. The alkene cleavage is the most prominent reaction catalysed by CiP, whereas MPO forms a larger amount of epoxide. The reaction was performed with a continuous and controlled flow of H_2_O_2_ (1 *μ*mol/h) to allow the formation of compound I and compound II (Scheme 1). The downside of employing such a low flow of oxidant is the long reaction time (16 h). The reaction mechanism of the alkene cleavage catalysed by CiP in presence of H_2_O_2_ as oxidant is different from the one previously described for CPO in presence of O_2_. In fact, the addition of a cosubstrate is not required in the case of the alkene cleavage catalysed by CiP using H_2_O_2_. Styrene (1 mM) was converted to styrene oxide (18%) and benzaldehyde (30%) employing CiP (20 *μ*M). Conversely, MPO furnished the epoxide as main product (17%) with only traces of the other compounds (5%). Activated substituted styrenes bearing chlorogroups in *orto-*, *meta-* and *para-*position and *cis-β*-methyl-styrene were converted as well, yielding to generally higher amounts of aldehydes.

## 3. Alkene Cleavage by Heme and ****Nonheme Oxygenases

Iron cofactor-dependent oxygenases constitute a very heterogeneous group of enzymes. Each enzyme family activates dioxygen in different manner, and it was also postulated that the same enzyme may cleave olefinic functionalities using different mechanisms depending on the substrate (e.g., carotenoid cleavage oxygenases). This property has been already reviewed for other enzymes, and it was named catalytic enzyme promiscuity [34–36]. The alkene cleavage is mainly a secondary activity for the peroxidases, whereas it is generally the natural and often unique activity for the oxygenases.

### 3.1. Tryptophan 2,3-Dioxygenase and Indoleamine 2,3-Dioxygenase

**Scheme 6:**
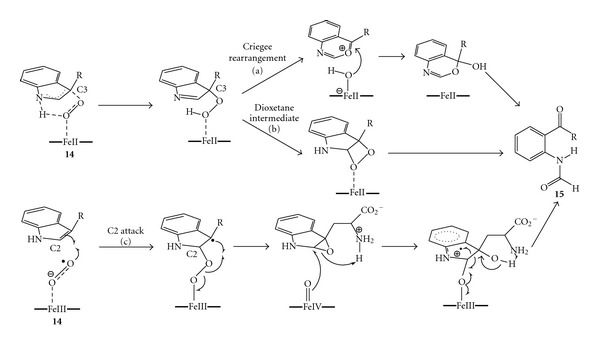
C=C double bond cleavage catalysed by tryptophan 2,3-dioxygenase (TDO). Historical perspective of the proposed reaction mechanisms: (a) C3-attack and Criegee rearrangement; (b) C3-attack and formation of dioxetane intermediate; (c) C2-attack via direct radical addition (current accepted mechanism).

Tryptophan 2,3-dioxygenase (TDO) and indoleamine 2,3-dioxygenase (IDO) are unique heme dependent dioxygenases which cleave the C=C double bond of the pyrrole ring of the tryptophan (**14**) to afford N-formylkynurenine (**15**) [37]; hence, both oxygen atoms are incorporated from molecular oxygen. TDO and IDO catalyse essentially the same reaction, with the different denominations merely reflecting the wider substrate promiscuity of IDO. Various reaction mechanisms for this intriguing alkene cleavage have been proposed since the 1930; yet a conclusive study has not been published so far. The first proposal involved the deprotonation of the aminogroup of the indole ring by the highly conserved histidine residue present in the active site of the enzyme [38]. In contrast, later studies demonstrated that methyltryptophane could also be cleaved by the enzyme whilst at reduced reaction rate [39]. Moreover site direct mutagenesis of the histidine residue to an alanine or a serine led to variants which still cleaved the natural substrate [39–41]. These findings are in agreement with the well-documented chemistry of indoles [42] which do not react by base-catalysed reaction (*PK*_*a*,N–H_ ≈ 17), thus ruling out the essential role of the histidine residue in a possible deprotonation step. The successive proposals encompassed the electrophilic addition of an activated Fe(II)-dioxo species to the C3 of the indole ring, followed by either Criegee-type rearrangement (Scheme 6, path (a)) or formation of a dioxetane intermediate (Scheme 6, path (b)). [39, 43]. On the contrary, the recent isolation and characterisation of a cyclic aminoacetal [44] (i.e., probably generated by the rearrangement of a 2-3-epoxide intermediate) coupled with novel Raman studies suggested a sequential insertion of oxygen [45]. A further computational study supported a mechanism whereby an Fe(III)-superoxo species (compound III, see Scheme 1) may be involved in a direct radical addition to the C2 of the indole ring, followed by the homolytic O–O cleavage and the formation of the 2-3 epoxide intermediate. In a second step, the Fe(IV)-oxo intermediate (compound II, see Scheme 1) might open the epoxide ring, attacking again the C2 of the indole ring, finally leading to the cleavage via an intramolecular rearrangement (Scheme 5, path (c)) [46]. It is noteworthy that the ultimate proposal does not require the initial deprotonation of the amino group of the indole. 

### 3.2. Catechol Dioxygenases

**Scheme 7:**
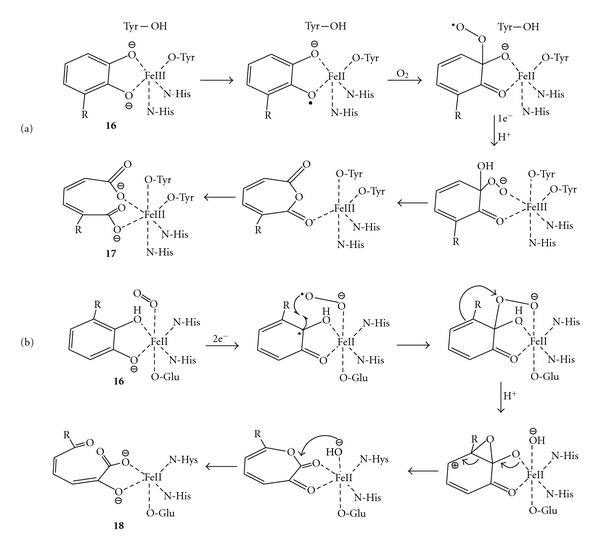
The mechanisms of the C=C double bond cleavage of catechols catalysed by (a) intradiol dioxygenase and (b) extradiol dioxygenase.

The catechol dioxygenases catalyse the oxidative cleavage of catechols and substituted catechols (**16**) as a central step in the bacterial degradation of aromatic compounds [47]. Hayaishi et al. discovered two nonheme families of these dioxygenases, namely, the intradiol dioxygenases (e.g., catechol 1,2-dioxygenase also called pyrocatechase) and the extradiol dioxygenases (e.g., catechol 2,3-dioxygenase also called metapyrocatechase). The intradiol dioxygenases cleave the C=C double bond between the phenolic hydroxyl groups to yield muconic acid (**17**) [48], whereas the extradiol dioxygenases cleave the C=C double bond adjacent to the phenolic hydroxyl groups to yield 2-hydroxymuconaldehyde (**18**) [49]. In the initial step, the intradiol dioxygenase interacts with molecular oxygen and probably generates an Fe(II)-semiquinone intermediate which then leads to Fe(III) species as confirmed by EPR studies. The successive steps, which end up to the C=C double bond cleavage of the aromatic ring, were previously believed to occur via a dioxetane intermediate; however, recent ^18^O_2_ experiments supported an alternative mechanism involving a Criegee rearrangement to furnish an anhydride intermediate which then hydrolyses to muconic acid (Scheme 7(a)) [50]. Similarly, the mechanism of alkene cleavage catalysed by extradiol dioxygenase proceeds initially again through an Fe(II)-semiquinone complex, although then diverging to form an Fe(II) proximal hydroperoxide intermediate; the latter undergoes to Criegee rearrangement followed by hydrolysis to afford 2-hydroxymuconaldehyde (Scheme 7(b)). Several findings supported this mechanism: (i) the analysis of the product distribution from the reaction with substrates analogues carrying a cyclopropyl radical [51], (ii) UV-visible and Raman spectroscopic studies [52] and (iii) ^18^O_2_ labelling studies [53]. 

### 3.3. Carotenoid Cleavage Oxygenases

**Scheme 8:**
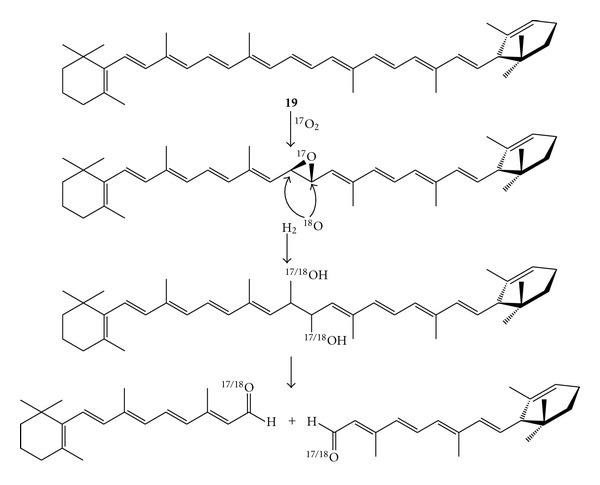
The proposed mechanism for the C15-C15′ double bond cleavage of *β*-carotene. Labelling experiment using ^17^O_2_ and ^18^H_2_O.

Carotenoid cleavage oxygenases (CCOs) are widespread in bacteria, plants, and animals and catalyse the C=C double bond cleavage of carotenoids to give apocarotenoids [54, 55]. The family members require an Fe(II) centre which is bound to four highly conserved and catalytically essential histidine residues [56]. CCOs often exhibit substrate promiscuity, which probably contributes to the natural diversity of apocarotenoids and derivates, whilst often retaining a perfect regioselectivity (i.e., cleavage of a specific C=C double bond of the carotenoid chain). Thus, depending on the enzyme, the alkene cleavage can occur either at the central C=C double bond of the carotenoid substrates (i.e., C15=C15′ position, central cleavage) or at another position (i.e., excentric cleavage). In the literature, this enzyme family is usually referred to as carotenoid cleavage dioxygenases (CCD). However, in this paper, the broader definition as carotenoid cleavage oxygenases (CCOs) was adopted since the classification as mono- or dioxygenases is still subject of debate within the scientific community. A monooxygenase enzyme activates molecular oxygen to incorporate one oxygen atom into the substrate, whereas the second oxygen originates from a water molecule. Conversely, a dioxygenase enzyme incorporates the two oxygen atoms coming from one molecule of molecular oxygen. Distinguishing between these two mechanisms relies on experiments using isotopically labelled O_2_ and H_2_O, which pose a serious problem in the analysis of the product distribution due to the rapid exchange of the aldehydic oxygen with the water medium. The controversial case of assignment of the cleavage mechanism for *β*-15-15′-carotenoid cleavage oxygenase (*β*-CCO) exemplifies this issue. The *β*-CCO from rat liver and rat intestine was initially termed as “*β*-carotene 15,15′-dioxygenase” albeit an evidence concerning a dioxygenase mechanism was not previously reported [57]. Later Leuenberger et al. claimed that the cleavage occurs via a monooxygenase activity [58]. *α*-Carotene (**19**) was chosen as substrate for the labelling studies, since only the use of a nonsymmetrical carotenoid giving different aldehydes may provide an exact information on the origin of the oxygen atoms incorporation. With the aim to minimise the oxygen scrambling between the product aldehydes and the medium, the experiment was carried out combining the *β*-CCO with a horse liver alcohol dehydrogenase (HL-ADH) so that the generated aldehydes were in situ reduced to the corresponding alcohols. The experiment was performed employing labelled ^17^O_2_ into labelled H_2_^18^O as reaction medium and the obtained product distribution was apparently consistent with a monooxygenase pathway, since equal amounts of ^17^O- and ^18^O-labelled aldehydes were revealed (Scheme 8).

**Scheme 9:**
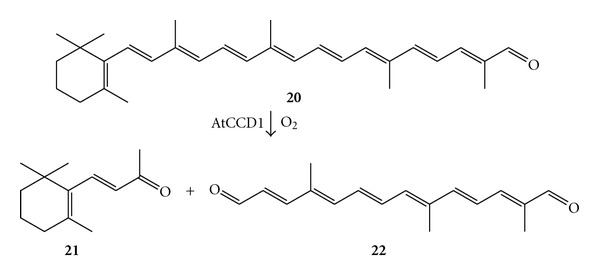
The C9–C10 double bond cleavage catalysed by the carotenoid cleavage dioxygenase from *Arabidopsis thaliana* (AtCCD1).

Despite the fact that *β*-CCO was reassigned as a *β*-carotene 15,15′-monooxygenase, several authors questioned about these results due to the long reaction time of the enzymatic reaction (7.5 h) and the dismutase activity of the HL-ADH, especially at increased level of NADH, which may lead to an unspecific water-derived oxygen incorporation into retinol [59]. In another study the reaction mechanism of the carotenoid cleavage oxygenase from *Arabidopsis thaliana* (AtCCD1) was investigated [60]. AtCCD1 cleaves *β*-apo-8′-carotenal (**20**) as natural substrate at the 9,10 double bond to deliver one molecule of *β*-ionone (**21**) and a molecule of C_17_ dialdehyde (**22**), (Scheme 9). 

In this case the very low rate for the exchange of oxygen atoms between the keto moiety of *β*-ionone and the medium coupled with a shorter reaction time (30 min) due to higher enzyme activity might provide an accurate evaluation of the reaction mechanism. When the experiment was performed in presence of ^18^O_2_, the obtained *β*-ionone was 96% labelled, whereas in the experiment in presence of ^18^H_2_O, the same product was completely unlabelled. The C_17_ dialdehyde underwent a partial oxygen exchange during the reaction time as supported by blank experiments; however, a significant fraction of C_17_ dialdehyde (27%) showed incorporation of one ^18^O atom when the experiment was performed in ^18^O_2_, hence supporting a dioxygenase mechanism. Furthermore, a recent computational study based on the crystal structure of AtCCD1 [61] estimated that the energy barrier for the formation of the epoxide intermediate (16.6 kcal mol^−1^) is only slightly exceeding the one for the dioxetane intermediate (15.9 kcal mol^−1^), probably due to the sterical effect of the Thr136 residue in the catalytic site [62]. Nevertheless a putative stilbene oxygenase sharing high sequence homology with the AtCCD1 was shown to cleave variously substituted stilbene derivatives via a monooxygenase mechanism (this enzyme will be discussed in the next session) [63]. Thus, subtle changes in the active site may favour one mechanism over the other or the same carotenoid cleavage oxygenase may display both reactivities depending on the substrate.

The products of the alkene cleavage of carotenoids and apocarotenoids constitute important natural flavours for the aroma industry; hence the possibility to exploit AtCCD1 in biocatalysis has attracted the interest of academia and industry. Schilling et al. reported an improved protocol for the expression of AtCCD1 in *E. coli* as a fusion protein with glutathione-S-transferase [64]. The recombinant enzyme showed higher level of heterologous expression as well as ameliorated biocatalytic performance. In the first report, an 18-fold increased activity in vitro was found when the enzymatic assay was conducted in a micellar dispersion using Triton X-100 as surfactant (substrate-surfactant, 0.008 ratio) and adding methanol as organic cosolvent (15% v v^−1^). Due to improved solvation of the lipophilic substrate in the aqueous micellar medium, *β*-apo-8′-carotenal was cleaved to give **β**-ionone with high conversion (>90%) and perfect regioselectivity. In a more recent publication it was shown that the maximum activity for *β*-apo-8′-carotenal varied dependently on the surfactant employed [65]. Testing diverse apocarotenoids and carotenoids in combination with different surfactants demonstrated that the most suitable surfactant varied dependently on the lipophilicity of the substrate. Nevertheless, the substrate concentration currently applied is too low (less than 1 mM) to meet the requirement for a possible industrial application.

### 3.4. Stilbene-**α*-*β**-Oxygenase and Isoeugenol Oxygenase

**Scheme 10:**
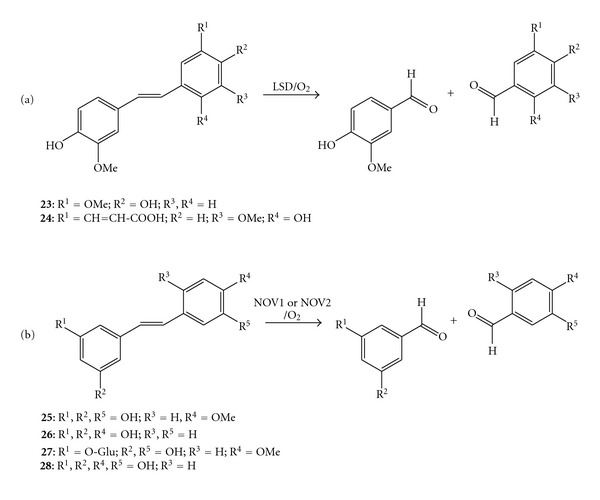
Alkene cleavage of stilbene derivates: (a) alkene cleavage catalysed by the isoenzymes of lignostilbene *α*,*β*-oxygenase from *Sphingomonas paucimobilis* TMY 1009; (b) alkene cleavage catalysed by stilbene monooxygenases from *Novosphingobium aromaticivorans* DSM 12444 NOV1 and NOV2.

Kamoda et al. identified and purified four isoenzymes of the stilbene-*α-β*-oxygenase (also named lignostilbene-*α*-*β*-dioxygenase, LSD) from *Sphingomonas paucimobilis* TMY 1009 (previously named *Pseudomonas p.*). This enzyme family was arbitrarily classified as dioxygenase, albeit studies to shed light on the mechanism were never undertaken. Therefore, the definition as oxygenase was adopted in this paper. The four enzymes contain one equivalent of iron, and they are constituted by two subunits: isoenzyme I (*αα*), isoenzyme II (*αβ*), isoenzyme III (*ββ*), and isoenzyme IV (*γγ*) [66, 67]. The catalytic activity of all isoenzymes required a stilbene-type substrate possessing *trans*-configuration and bearing a hydroxylic substituent in *para-*position on the aromatic ring. The four isoenzymes cleaved 4,4′-dihydroxy-3,3′-dimethoxystylbene (**23**) as well as 4,2′-dihydroxy-3,3′-dimethoxy-5′-(2′′-carboxyvinyl)-stilbene (**24**), although showing different substrate specificities (Scheme 10(a)); the isoenzyme I was the most active enzyme with a preference for **23**, whereas the others cleaved preferentially substrate **24** [68]. Particularly, the isoenzyme I was stable at 50 °C and showed increased activity upon the addition of methanol as cosolvent (30% v v^−1^) [69]. Despite the fact that the cleavage of compound **23** can furnish directly two molecules of vanillin, an important flavour and fragrance for the food and cosmetic industry, the enzymatic reaction was never exploited on a preparative scale. A recent survey of the bacterial genome sequence for carotenoid cleavage oxygenase homologues allowed to identify two putative stilbene oxygenases from *Novosphingobium aromaticivorans* DSM 12444 (NOV1 and NOV2) [63]. NOV1 and NOV2 cleaved selectively *trans*-stilbene-type substrates bearing a hydroxy- or a methoxy moiety in *para*-position of the phenyl ring such as rhapontigenin (**25**), resveratrol (**26**), rhaponticin (**27**), and piceatannol (**28**), (Scheme 10(b)). Interestingly, the two enzymes were not able to cleave carotenoids. Labelling studies using ^18^O or ^18^H_2_O showed that NOV1 and NOV2 incorporated only one oxygen atom from molecular oxygen into the substrate. Thus the two putative stilbene oxygenases were classified as monooxygenases. It is interesting to note that AtCCD1 was classified as dioxygenase while NOV1 and NOV2 as monooxygenases in spite of high sequence homology.

**Scheme 11:**
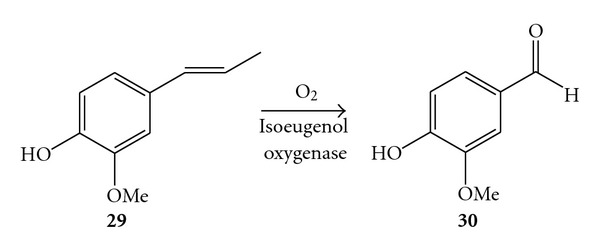
Alkene cleavage catalysed by isoeugenol oxygenase from *Pseudomonas putida* IE27.

In another study, a novel enzyme was unveiled from *Pseudomonas putida* IE27 when the microorganism was cultivated from soils containing isoeugenol as a sole carbon source [70]. The enzyme was purified and assayed for the catalytic reaction in vitro, demonstrating high activity for the alkene cleavage of isoeugenol (**29**) to yield vanillin (**30**) in strict presence of molecular oxygen (Scheme 11). Besides, 2-methoxy-4-vinylphenol was also a substrate albeit at a reduced reaction rate (i.e., two orders of magnitude). Hence the enzyme was named after its substrate as isoeugenol oxygenase. Interestingly the analysis of the amino acid sequence of the isoeugenol oxygenase revealed high homology with the previously mentioned stilbene-*α*-*β*-oxygenase from *Sphingomonas paucimobilis* TMY 1009 isoenzyme I and III (42% identity) and a putative stilbene oxygenase from *Novosphingobium aromaticivorans* DSM 12444 (40% identity). The alkene cleavage of isoeugenol was postulated to occur through a monooxygenase mechanism on the base that vanillin incorporated a labelled ^18^O atom when the reaction was carried out either in labelled ^18^O_2_ or ^18^H_2_O. Nonetheless the monooxygenase mechanism cannot be considered as conclusive, due to the lack of data about isotopic product distribution; data about exchange of oxygen atoms from substrate to the aqueous medium were not reported as well.

### 3.5. Other Oxygenases

**Scheme 12:**
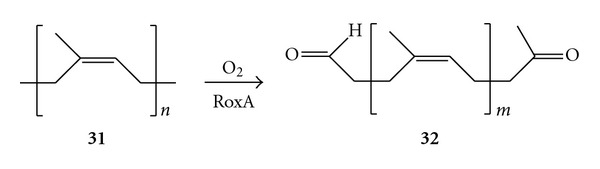
Alkene cleavage of natural rubber catalysed by the rubber oxygenase (RoxA) from *Xanthomonas sp*. The major product showed *m* = 2.

Other putative oxygenases have been isolated acting on various substrates. In this last paragraph, the most promising enzymes for biocatalytic applications will be examined. The enzymatic C=C double bond cleavage of natural rubber (i.e., poly-(*cis*-1,4-isoprene)) (**31**) and synthetic rubbers was observed using a purified protein from *Xanthomonas *sp., currently named as rubber oxygenase (RoxA), (Scheme 12) [71]. RoxA was characterised with the aid of UV-visible spectroscopy and gene sequence analysis, revealing two heme prosthetic groups located into protein scaffold and a conserved sequence motif which is a distinctive feature of the cytochrome *c* peroxidases. The enzymatic activity strictly necessitated molecular oxygen and completely ceased when heme inhibitors such as potassium cyanide and carbon monoxide were added into the reaction mixture, thus confirming the essential catalytic role of the metal centre. RoxA showed high regioselectivity since it was capable to cleave poly-(*cis*-1,4-isoprene) at regular intervals, principally cutting off three isoprene units per step (**32**). A limited amount of major and minor degradation products was also detected, however always showing a carbonylic moiety at both terminal ends. Further labelling studies revealed RoxA to cleave following a dioxygenase mechanism [72].

**Scheme 13:**
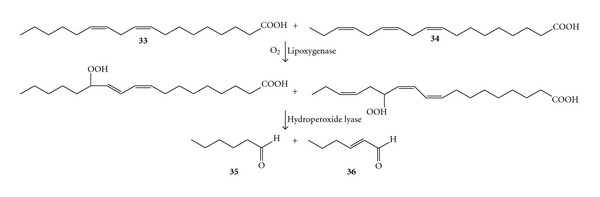
Formal alkene cleavage of polyunsaturated fatty acids combining a lipoxygenase and a hydroxyperoxide lyase.

Two enzymes can also cooperate to achieve the alkene cleavage. For instance, in the first step, the lipoxygenase from soybean flour activated molecular oxygen to enable the attack of the olefinic group. In the second step the hydroperoxide intermediate was cleaved by the fatty acid hydroperoxide lyase (HPO lyase) from green bell pepper. Thus, the formal two-step cleavage of a mixture of linoleic (**33**) and linolenic acids (**34**) to yield hexanal (**35**) and *trans*-hexenal (**36**), respectively, was carried out on a preparative scale with a productivity of ca. 300 mg L^−1^ (Scheme 13). The C6 aldehyde products are important flavours for the aroma industry [73]. 

**Scheme 14:**
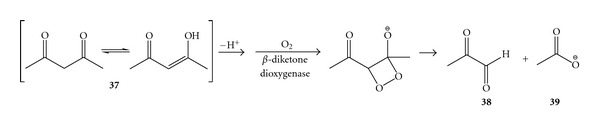
Alkene cleavage catalysed by *β*-diketone dioxygenase.

Finally, even a formally single C–C bond can be cleaved following a dioxygenase mechanism. That is the case of the Fe(II)-dependent *β*-diketone dioxygenase from *Acinetobacter johnsonii* (Dke1). Dke1 cleaves the enol form of the 2,4-pentanedione (**37**) (plus related *β*-dicarbonyl compounds thereof), giving equimolar amounts of methylglyoxal (**38**) and acetate (**39**) and consuming only one equivalent of molecular oxygen (Scheme 14) [74].

## 4. Conclusions and Outlook

While studying heme peroxidases to carry out chemical transformations such as the asymmetric epoxidation or the stereoselective mono- and dihydroxylation of unsaturated functionalities, the alkene cleavage has been revealed as a minor side reaction. In few cases, the alkene cleaving activity became the predominant one when the reaction conditions were properly adjusted (i.e., pH, dioxygen pressure, addition of cosolvents). Thus, this promiscuous activity can be potentially exploited in organic synthesis. In contrast, iron-dependent oxygenases often catalyse the alkene cleavage as the natural and unique reaction. Yet, the exploitation of these enzymes in organic synthesis is hampered by the limited solubility of apolar substrates in aqueous media. 

On the other hand, especially in the case of heme peroxidases, a comprehensive understanding of the reaction mechanisms for the alkene cleavage necessitates more accurate studies. In fact, the proposed mechanisms are merely based on experimental observations, that is, analysis of the product distribution, requirement of dioxygen and cosubstrates, and so forth, or rely on studies of selective inhibition of the heme cofactor. 

A detailed understanding of the alkene cleaving mechanism coupled with the advancement in gene cloning and protein engineering over the last decade will allow us to manipulate these enzymes to ameliorate their chemo- and regioselectivity and increase their tolerance to harsher reaction conditions. The design of improved variants could finally pave the way to the application of the enzymatic alkene cleavage on a large scale.
